# Clinician ratings of the Comprehensive Assessment of Psychopathic Personality (CAPP) in a representative sample of Spanish prison inmates: New validity evidence

**DOI:** 10.1371/journal.pone.0195483

**Published:** 2018-04-12

**Authors:** Gerardo Flórez, Ventura Ferrer, Luis S. García, María R. Crespo, Manuel Pérez, Pilar A. Saíz, David J. Cooke

**Affiliations:** 1 Centro de Investigación Biomédica en Red de Salud Mental (CIBERSAM), Oviedo, Spain; 2 Health Department, Pereiro de Aguiar Prison, Ourense, Spain; 3 Department of Psychiatry, University of Oviedo, Oviedo, Spain; 4 Department of Psychosocial Science, University of Bergen, Bergen, Norway; Universite de Bretagne Occidentale, FRANCE

## Abstract

The Comprehensive Assessment of Psychopathic Personality (CAPP) is a concept map of psychopathic personality disorder (PPD). The CAPP- Institutional Rating Scale (IRS) is a tool designed to assess CAPP symptoms in institutional settings. The CAPP contains 33 personality traits organized in six domains: attachment, behavioural, cognitive, dominance, emotional and self. Until now, much of the CAPP research has been conducted out of clinical, forensic and correctional settings using self-ratings. In the current study, the psychometric properties and construct validity of the CAPP-IRS were evaluated in a non-convenience sample of 204 Spanish convicts. Clinician ratings were employed. Participants had been imprisoned for at least 6 months at Pereiro de Aguiar Penitentiary. This group of inmates was heterogeneous with respect to type of official charges, and representative as all convicts interned for at least 6 months in this prison were screened for participation. Classical test theory indexes of reliability, correlations between CAPP items and domains and external correlations and structural analyses demonstrated that CAPP assessment is a solid and robust way of evaluating psychopathy in a correctional setting. Best fit was found for a three-factor model: attachment and emotional items associated with a callous and unemotional trait, dominance and self items associated with a pathological interpersonal style, and behavioural and residual items from other domains associated with impulsivity.

## Introduction

Psychopathic Personality Disorder (PPD) or Psychopathy is a severe personality disorder characterized by the following traits: callous and unemotional (CU) affects reflecting a deficient affective experience (*affective processing*), grandiose and arrogant interpersonal style (*Interpersonal antagonism*), and pervasive impulsive conduct (*behavioural dysfunction*) [1–4] When followed-up, these individuals experience more adverse outcomes when compared with the general population, such as a disproportionate amount of crime and violence, and poor treatment response that leads to recidivism [1, 5–8] With this in mind, it becomes obvious why clinical and forensic staff require assessment procedures for the detailed description and diagnosis of psychopathy. Such reliable assessment tools must reduce the risk of false positives and negatives to a minimum, as an incorrect diagnosis can have detrimental consequences for society (false negative) or for the person being assessed (false positive) [2, 9, 10] To achieve this accuracy, these tools must assess the traits that are at the core of the disorder [11, 12] Unfortunately, scholars and researchers are still debating this issue, especially wether affective & interpersonal traits are sufficient for establishing that someone has PPD or whether behavioural traits mainly criminal and antisocial are also needed [2–4, 9, 11, 13–21].

Blackburn [22] cogently argued that if we are to understand the association between personality pathology and criminal behaviour, it is essential to measure both separately. Thus, a measure of psychopathy that evaluates personality pathology independent of criminal history should be of great value to researchers trying to resolve the debate about the diagnostic significance of antisocial and criminal behaviour.

Cooke and colleagues developed the *Comprehensive Assessment of Psychopathic Personality* (CAPP) [23, 24] The CAPP is a concept map based on a dynamic personality trait approach [25] The focus is on personality traits and its pathology rather than on specific behaviours also known as characteristic adaptations [26] Founded on the lexical hypothesis, which states that salient dimensions of personality variation are richly represented in natural language, a concept map containing 33 personality traits was derived. The goal was to be comprehensive in the coverage of all the primary symptoms of PPD, and to describe these symptoms at the basic level of discrete features of personality [27] The CAPP concept map provides the underpinning of different measures: the distinction between measures is important to avoid operationalism. The CAPP- Institutional Rating Scale (IRS) was developed as an expert rating scale suitable for use in clinical and forensic settings. The symptoms within the CAPP are organised into six conceptual domains (*Attachment*, *Behavioural*, *Cognitive*, *Dominance*, *Emotional*, and *Self*) [25] This gives the CAPP a hierarchical structure. Seven-point scale ratings of each symptom are given for an assessment timescale that usually ranges from 6 to 12 months, so that change over time in the nature and severity of PDD can be assessed [27] Data are collected through a semi-structured interview, using a simple and open questioning style, file and collateral information provided by informants with extensive knowledge of the client.

The CAPP concept map was developed using a multi-modal “bottom-up” approach. The following steps were taken: review of the literature to find PPD primary symptoms, expert consultation on the identified symptoms, definition of the symptoms in terms of trait-descriptive adjectives (three trait-descriptive adjectives were used for each symptom), and categorization of the symptoms into distinct domains of psychological functioning [25, 27].

The robustness and relevance of the CAPP model has been tested across languages, experts and lay people, and sex through prototypicality analysis [23, 24, 28, 29] Research has also shown that CAPP content validity is high. Best fit has been reported for a model with one general factor representing *global psychopathy*, and three residual factors: *boldness/emotional stability*, *emotional detachment*, and *disinhibition [30]* These were not clinician-rating studies but mostly self-ratings ones. In terms of predictive validity for reoffending, the CAPP has shown promising results compared with other instruments [31].

Studying the CAPP psychometric properties and construct validity in a non convenience sample of participants can help elucidate the previously mentioned antisocial debate. The use of clinician-ratings in a non-convenience inmate sample can help clarify whether previous promising results from non-clinical or forensic studies with the CAPP can be generalized to settings in which the CAPP has a potential to be used.

### Objectives

The current study was designed to examine CAPP scores in a sample of inmates from Pereiro de Aguiar Penitentiary (in Spain) in order to validate the Spanish version of the CAPP and to clarify its construct validity. The main hypothesis was that the CAPP model would be as robust and relevant for assessing a sample of inmates as it proved to be in previous prototypicality and self-rating studies.

## Materials and methods

### Participants

The study was conducted at Pereiro the Aguiar Penitentiary, a low-medium security institution where all offenders from the Ourense region who receive aggregate sentences of 2 years or longer are imprisoned. The prison also accepts inmates from other Spanish regions and prisons. All inmates incarcerated between April 2014 and April 2016 were screened. Inclusion criteria were: having served at least 6 months of their sentence at the Pereiro de Aguiar Penitentiary, a requirement from the CAPP protocol implemented to improve the quality of the collateral information provided by the penitentiary staff, and providing written informed consent. Exclusion criteria were: not being a fluent Spanish speaker or having a diagnosis of a serious neurological or psychiatric disorder.

In all, 330 inmates were screened. Of those, 126 (38.18%) did not meet the inclusion-exclusion criteria: 10 (7.93%) refused to participate and did not sign the written informed consent, 16 (12.69%) were not fluent Spanish speakers, 32 (25.39%) have been diagnosed with a serious neurological or psychiatric condition (15 with schizophrenia and related disorders, 10 with mayor affective disorders, and 7 with neurological cognitive impairment), and 68 (53.99%) had not served at least 6 months of their sentence at the prison. Thus, 204 (61.82%) inmates fulfilled the inclusion and exclusion criteria and were enrolled in the study. Then, of the 262 prisoners who fulfilled the inclusion criteria, 204 (77.82%) participated in the study, and only 10 (3.81%) refused to do so.

The protocol was approved by the Pontevedra-Vigo-Ourense Local Research Ethics Committee (2014/009), and every participant provided written informed consent. The study was conducted in accordance with the Declaration of Helsinki. No financial or other compensation was offered. Participants in the study were able to opt out whenever they wanted to do so. As there was no research treatment in the study, all inmates, whether participants or not, received the same treatments.

### Procedure

All participants completed the following protocol:

- CAPP: One of the researchers, GF, trained and experienced in the use of the CAPP, interviewed all participants and coded the scores. It should be noted that during the entire interview he was blind to the sociodemographic and forensic data during the entire interview.
- International Personality Disorder Examination (IPDE) DSM version: One of the researchers, GF, trained and experienced in the use of the IPDE, interviewed all participants and coded the scores, once again blinded to the sociodemographic and forensic data. Categorical personality disorders diagnoses were collected to study their relationship to CAPP scores and also to have a measure for comparison with other inmate population samples [32, 33].
- Sociodemographic and forensic variables: The following variables were collected by researchers other than GF, blinded to the CAPP and IPDE scores: Sex, age, nationality, number of education years completed, marital status, total monthsin prison, current drug / alcohol use (type, age of first use, principal route of administration) and type of official charges. DSM-IV-TR diagnoses were used.

Table 1 provides a summary of the IPDE scores and sociodemographic and forensic variables of the sample.

**Table 1:** IPDE scores, sociodemographic and forensic variables of the sample. Levels of significance for these variables in relation to an arbitrary CAPP cut-off score of 76 (the sample mean) are also included.

Variables	% of inmates	CAPP of 76 or more (n = 104)
Sex		
Male	176 (86.27%)	85 (41.67%)
Female	28 (13.73%)	19 (9.31%)
		X^2^ = 3.699 p = 0.054446
Age (mean (SD))	40.93 (11.18)	40.6 (11.80)
		T = 0.47 p = 0.640
Nationality		
Spanish	179 (87.75)	91 (44.61%)
Others	25 (12.25)	13 (6.37%)
		X^2^ = 0.0119 p = 0.913306
Education years completed (mean, (SD))		
Basic	8.84 (1.95)	8.68 (1.93) T = 1.04 p = 0.299
Higher	0.24 (0.88)	0.250 (0.833) T = -0.25 p = 0.803
Marital status		
Married	49 (24.01)	18 (8.82%)
Separated /divorced	61 (29.9)	35 (17.16%)
Widowed	1 (0.51)	1 (0.49%)
Single	93 (45.58)	50 (24.51%)
		p = 0.0085
Total months in prison (mean (SD))	75.08 (83.56)	89.5 (82.9)
		T = -2.54 p = 0.012
Drug / Alcohol use		
Alcohol	165 (80.88)	83 (40.69%) X^2^ = 0.046
		p = 0.830218
Alcohol abuse	78 (38.24)	34 (16.67%) X^2^ = 2,7602 p = 0.096634
Heroin	90 (44.12)	63 (30.88%) X^2^ = 23.313 p = 0.000001
Methadone	70 (34.31)	49 (24.02%) X^2^ = 15.426 p = 0.000086
Other Opiates	15 (7.35)	12 (5.88%) p = 0.029277
Benzodiazepines	38 (18.63)	27 (13.24%) X^2^ = 7.5288 p = 0.006072
Cocaine	125 (61.27)	72 (35.29%) X^2^ = 6.3498 p = 0.011739
Amphetamines	28 (13.73)	18 (8.82%) X^2^ = 3.064 p = 0.080045
Cannabis	117 (57.35)	71 (34.80%) X^2^ = 11.2535 p = 0.000795
Hallucinogens	30 (14.71)	21 (10.29%) X^2^ = 6.2144 p = 0.012672
Inhalants	7 (3.43)	7 (3.43%) p = 0.0141692
Two or more	142 (60.61)	89 (43.63%) X^2^ = 9.7779 p = 0.001766
Three or more	112 (54.90)	75 (36.76%) X^2^ = 6.4604 p = 0.01103
Four or more	92 (45.10)	64 (31.37%) X^2^ = 10.3598 p = 0.001288
Two or more (neither alcohol nor methadone)	114 (55.88)	72 (35.29%) X^2^ = 14.2626 p = 0.000159
Three or more (neither alcohol nor methadone)	86 (42.16)	58 (28.43%) X^2^ = 16.1217 p = 0.000059
Four or more (neither alcohol nor methadone)	49 (24.02)	37 (18.14%) X^2^ = 15.5278 p = 0.000081
Type of official charges		
Drug dealing	79 (38.73)	44 (21.57%) X^2^ = 1.1473 p = 0.28411
Crimes against property	116 (56.86)	68 (33.33%) X^2^ = 6.2814 p = 0.012202
Violent crimes	91 (44.61)	52 (25.49%) X^2^ = 2.4965 p = 0.114101
Other crimes	54 (26.47)	31 (35.78%) X^2^ = 1.2139 p = 0.270564
Disorderly conduct	32 (15.69)	22 (10.78%) X^2^ = 4.7955 p = 0.028534
Driving while intoxicated	42 (20.59)	30 (14.71%) X2 = 9.765 p = 0.0018
Major traffic violations	60 (29.41)	13 (6.37%) X^2^ = 8.4892 p = 0.003573
Two or more	149 (73.04)	79 (38.73%) X^2^ = 0.9201 p = 0.337451
Three or more	81 (39.71)	48 (23.53%) X^2^ = 3.6845 p = 0.05492
Four or more	26 (12.75)	17 (8.33%) X^2^ = 2.4739 p = 0.115747
IPDE diagnosis		
Paranoid	29 (14.22)	26 (12.75%) p = 0.0000040256
Schizoid	0 (0)	0 (0%) p = 1
Schizotypal	1 (0.49)	1 (0.49%) p = 1
Antisocial	38 (18.63)	33 (16.18%) X^2^ = 24.0323 p = 0.000001
Borderline	15 (7.35)	12 (5.88%) p = 0.0292772459
Histrionic	13 (6.37)	10 (4.90%) p = 0.0828034
Narcissistic	43 (21.08)	39 (19.11%) p<0.000000001
Avoidant	17 (8.33)	9 (4.41%) X^2^ = 0,0285 p = 0.865865
Dependent	2 (0.98)	0 (0%) p = 0.2390611
Obsessive	2 (0.98)	2 (0.98%) p = 0.49773
More than one	103 (50.49)	41 (20.10%) X^2^ = 27.5825 p = 0.000001

CAPP: Comprehensive Assessment of Psychopathic Personality; SD: Standard Deviation.

### Analyses

The study variables were described as means of average values and standard deviations for continuous variables and percentages for categories. Independent sample t-tests were performed to determine if there were differences among groups. For categorical variables the chi-square test was used for the same purpose. The strength and direction of linear relations among variables was determined with the Pearson correlation coefficient while Spearman’s rank-order correlation was also used for those cases in which both variables were ranked. The CAPP scale was analysed with the help of a multivariate regression analysis. A Principal Component Analysis was conducted in order to perform a dimensional reduction and explore relationships among variables. Cronbach's alpha was used as a common measure of internal consistency (a measure of reliability). The reliability was also measured with the help of Classical Test Theory indexes including mean inter-item correlation and corrected item total correlation. Finally, Confirmatory Factor Analysis and Exploratory Bi-Factor Analysis were also performed to compare different model factor structures. The Exploratory Bi-Factor Analysis is an alternative structural model in which the covariance of all items is presumed to be explained both by general factors that reflect the overlap across all items and by separate uncorrelated grouping factors that reflect the unique coherency among particular subgroups of items. Several measures of model fit were used: chi-square, Comparative (Fit) Index (CFI), Tucker-Lewis Index (TLI) and RMSEA (Root Mean Square Error of Approximation). As in previous research (Storey, Hart, Cooke, & Michie, 2015) the criteria for adequate fit were defined as follows: CFI≥0.9 and RMSEA≤0.08.

## Results

Table 1 indicates that CAPP total score does not seem to be related to: Sex, age, education, or nationality, but does seem to be related to: Marital status, total months in prison, use of alcohol/drugs (in general, more use means a higher CAPP score), type of official charges (crimes against property and disorderly conduct, driving while intoxicated, and major traffic violations related positively) and IPDE diagnoses (paranoid, antisocial, borderline, and narcissistic showed a positive association). For purposes of comparison, an arbitrary cut-off score of 76 was used. It was chosen because it was the sample´s mean; 104 inmates (50.98%) scored 76 or more.

Correlations were calculated for all variables in Table 1 in relation to the CAPP dimensions and total score. The following correlations were highly significant: IPDE paranoid and attachment (0.443, p< 0.001), cognitive (0.359, p< 0.001), dominance (0.381, p< 0.001), emotional (0.398, p< 0.001), and total (0.386, p< 0.001); IPDE antisocial and behavioural (0.42, p< 0.001), cognitive (0.377, p< 0.001), dominance (0.354, p< 0.001), emotional (0.348, p< 0.001), and total (0.385, p< 0.001); IPDE narcissistic and attachment (0.365, p< 0.001), cognitive (0.265, p< 0.001), dominance (0.479, p< 0.001), emotional (0.362, p< 0.001), self (0.669, p< 0.001), and total (0.478, p< 0.001); Number of IPDE diagnosis and attachment (0.478, p< 0.001), behavioural (0.502, p< 0.001), cognitive (0.562, p< 0.001), dominance (0.596, p< 0.001), emotional (0.572, p< 0.001), self (0.67, p< 0.001), and total (0.65, p< 0.001).

Table 2 shows the distribution of CAPP total, dimension and item scores. Table 2 also presents Cronbach's alpha scores for the CAPP. Internal consistency was excellent for all domains and for total score.

**Table 2:** Distribution of CAPP total, domains and items. Cronbach’s alpha calculated without the specified variable.

**Variable**	**Mean (SD)**	**Cronbach´s alpha**
**Total**	**84.43 (42.02)**	**0.9598**
**Attachment Domain**	**9.559 (5.78)**	**0.9526**
Attachment Item 18 Detached	2.652 (1.754)	0.9593
Attachment Item 8 Uncommitted	2.647 (1.595)	0.9576
Attachment Item 25 Unempathic	2.01 (1.716)	0.9581
Attachment Item 24 Uncaring	2.25 (1.646)	0.9574
**Behavioural Domain**	**12.412 (8.281)**	**0.9538**
Behavioural Item 3 Lacks Perseverance	1.863 (1.822)	0.9589
Behavioural Item 26 Unreliable	2.358 (1.829)	0.9583
Behavioural Item 15 Reckless	2.539 (1.683)	0.9592
Behavioural Item 6 Restless	2.108 (1.722)	0.9593
Behavioural Item 17 Disruptive	1.936 (1.628)	0.9583
Behavioural Item 32 Aggressive	1.608 (1.545)	0.9582
**Cognitive Domain**	**10.191 (5.815)**	**0.9552**
Cognitive Item 19 Suspicious	3.181 (1.756)	0.9593
Cognitive Item 28 Lacks concentration	1.402 (1.617)	0.9599
Cognitive Item 7 Intolerant	1.466 (1.533)	0.9581
Cognitive Item 27 Inflexible	2 (1.538)	0.8583
Cognitive Item 29 Lacks planfulness	2.142 (1.913)	0.9590
**Dominance Domain**	**24.721 (12.666)**	**0.9495**
Dominance Item 11 Antagonistic	1.2696 (1.4075)	0.9580
Dominance Item 12 Domineering	1.76 (1.654)	0.9582
Dominance Item 10 Deceitful	2.505 (1.689)	0.9583
Dominance Item 9 Manipulative	2.632 (1.645)	0.9580
Dominance Item 23 Insincere	2.775 (1.642)	0.9582
Dominance Item 30 Garrulous	2.108 (1.609)	0.9590
**Emotional Domain**	**11.672 (5.926)**	**0.9543**
Emotional Item 5 Lacks Anxiety	2.779 (1.624)	0.9585
Emotional Item 33 Lacks Pleasure	1.569 (1.547)	0.9601
Emotional Item 4 Lacks Emotional Depth	1.819 (1.676)	0.9593
Emotional Item 31 Lacks Emotional Stability	2.074 (1.73)	0.9579
Emotional Item 16 Lacks Remorse	3.431 (1.67)	0.9580
**Self Domain**	**15.887 (9.219)**	**0.9504**
Self Item 20 Self-centred	2.887 (1.973)	0.9577
Self Item 14 Self-aggrandising	1.897 (1.757)	0.9585
Self Item 1 Sense of Uniqueness	2.333 (1.859)	0.9585
Self Item 13 Sense of Entitlement	2.118 (1.686)	0.9584
Self Item 22 Sense of Invulnerability	1.966 (1.732)	0.9581
Self Item 2 Self-justifying	3.377 (1.628)	0.9586
Self Item 21 Unstable Self-concept	1.299 (1.647)	0.9602

CAPP: Comprehensive Assessment of Psychopathic Personality; SD: Standard Deviation.

Table 3 shows reliability indexes from Classical Test Theory (CTC) as mean inter-item correlation and corrected-item total correlation. These index ´results were also excellent for all CAPP domains and total score.

**Table 3:** Reliability indexes from Classical Test Theory: CAPP total and dimension scores.

CAPP score	N	Alpha	MIC	CITC: Mdn (range)
CAPP Total	204	0.96	0.4195	0.670 (0.398; 0.804)
CAPP Attachment	204	0.88	0.6565	0.741 (0.525; 0.804)
CAPP Behavioural	204	0.89	0.5827	0.631 (0.530; 0.685)
CAPP Cognitive	204	0.73	0.3594	0.569 (0.445; 0.693)
CAPP Dominance	204	0.88	0.5526	0.687 (0.575; 0.726)
CAPP Emotional	204	0.77	0.3926	0.645 (0.407; 0.738)
CAPP Self	204	0.87	0.4813	0.651 (0.398; 0.757)

CAPP: Comprehensive Assessment of Psychopathic Personality; MIC: mean inter-item correlation; CITC: corrected-item total correlation; Mdn: median.

For the CAPP regression analysis model, the following variables were significant at an alpha level of 5% for the whole sample: IPDE paranoid (F = 32.16, p <0.0001), IPDE antisocial (F = 4.09, p = 0.045), IPDE borderline (F = 7.09, p = 0.009), and IPDE narcissistic (F = 16, p <0.0001).

Table 4 shows correlations among CAPP (Total and Domain scores) for the whole sample. The lowest correlation was between attachment and behavioural (0.55), and the highest between dominance and emotional (0.91). Actually, behavioural showed the lowest correlation not only with attachment but also with dominance (0.63), emotional (0.61), and self (0.59). Therefore, behavioural was the domain with the lowest correlations of all, and the correlation between the other domains scored higher than 0.65.

**Table 4:** Correlations among CAPP total and domain scores for the whole sample.

	CAPP Attachment	CAPP Behavioural	CAPP Cognitive	CAPP Dominance	CAPP Emotional	CAPP Self	CAPP Total
CAPP Attachment	**1.00**						
CAPP Behavioural	0.55^*^	**1.00**					
CAPP Cognitive	0.70^*^	0.75^*^	**1.00**				
CAPP Dominance	0.80^*^	0.63^*^	0.78^*^	**1.00**			
CAPP Emotional	0.79^*^	0.61^*^	0.78^*^	0.91^*^	**1.00**		
CAPP Self	0.68^*^	0.59^*^	0.67^*^	0.84^*^	0.73^*^	**1.00**	
CAPP Total	0.84^*^	0.78^*^	0.87^*^	0.96^*^	0.91^*^	0.88^*^	**1.00**

CAPP: Comprehensive Assessment of Psychopathic Personality

* p<0.001

Confirmatory factor analysis (CFA) was applied to CAPP scores using the Lavaan package[34] of the R statistical software. Weighted least-squares and maximum-likelihood estimation were used. The following exploratory models were compared: All dimensions, without cognitive, without behavioural, without behavioural and cognitive, without behavioural & cognitive & emotional, and without behavioural & cognitive& emotional & dominance, and a three-factor model (attachment and emotional versus behavioural and cognitive versus dominance and self). None of the fits can be considered to correspond to a good model (Table 5).

**Table 5:** CAPP Confirmatory factor analysis and bi-factor EFA.

Model	N	df	CFI	TLI	RMSEA
*CFA Models*					
**Weighted least squares**					
CAPP unidimensional	204	489	0.825	0.811	0.198
CAPP without Behavioural domain	204	319	0.876	0.863	0.174
CAPP without Cognitive domain	204	372	0.719	0.694	0.136
CAPP without Behavioural and Cognitive domains	204	528	0.662	0.634	0.14
CAPP without Behavioural & Cognitive & Emotional domains	204	75	0.834	0.799	0.160
CAPP without Behavioural & Cognitive& Emotional & Dominance domains	204	88	0.835	0.803	0.150
Three-factor	204	317	0.803	0.782	0.211
**Maximum likelihood**					
CAPP unidimensional	204	489	0.661	0.634	0.140
CAPP without Behavioural domain	204	319	0.703	0.673	0.140
CAPP without Behavioural and Cognitive domains	204	528	0.825	0.810	0.199
CAPP without Behavioural & Cognitive & Emotional domains	204	75	0.724	0.654	0.145
CAPP without Behavioural & Cognitive& Emotional & Dominance domains	204	88	0.723	0.658	0.111
Three-factor	204	317	0.676	0.641	0.148
*Bi-Factor EFA Models*					
Three bi-factors	204	402	0.968	0.959	0.075

CAPP: Comprehensive Assessment of Psychopathic Personality; S-BX2: chi-square, df: degrees of freedom; CFI: Comparative (Fit) Index; TLI: Tucker-Lewis Index; RMSEA: Root Mean Square Error of Approximation.

p<0.001 for all models.

Next, exploratory bi-factor models were evaluated, showing best fit for one general PPD factor and between 3 oblique bi-factors in keeping with previous research [30]. Table 5 also presents the model fit statistics associated with these models, and Table 6 shows the Fully Standardized Factor Loadings for Bi-Factor EFA Model.

**Table 6:** Fully Standardized Factor Loadings for Bi-Factor EFA Model.

Bi-Factor EFA Model				
CAPP Items	λGeneral	λDetachment	λDisinhibition	λDeceitfulnesss
Attachment Item 18 Detached	**0.608**	**0.535**	-0.220	-0.013
Attachment Item 8 Uncommitted	**0.779**	0.208	0.163	0.065
Attachment Item 25 Unempathic	**0.804**	**0.321**	-0.109	0.005
Attachment Item 24 Uncaring	**0.815**	0.268	0.205	0.046
Behavioural Item 3 Lacks Perseverance	**0.488**	0.124	**0.758**	0.210
Behavioural Item 26 Unreliable	**0.597**	0.039	**0.641**	0.273
Behavioural Item 15 Reckless	**0.469**	-0.289	**0.667**	-0.100
Behavioural Item 6 Restless	**0.508**	**-0.368**	**0.580**	-0.282
Behavioural Item 17 Disruptive	**0.632**	-0.171	**0.606**	-0.105
Behavioural Item 32 Aggressive	**0.769**	0.056	0.120	-0.257
Cognitive Item 19 Suspicious	**0.609**	**0.335**	-0.246	0.087
Cognitive Item 28 Lacks concentration	**0.357**	-0.036	**0.759**	-0.007
Cognitive Item 7 Intolerant	**0.826**	0.141	-0.044	-0.227
Cognitive Item 27 Inflexible	**0.746**	0.179	0.024	-0.283
Cognitive Item 29 Lacks planfulness	**0.469**	0.065	**0.768**	0.064
Dominance Item 11 Antagonistic	**0.812**	0.150	0.086	-0.187
Dominance Item 12 Domineering	**0.842**	0.000	-0.251	-0.186
Dominance Item 10 Deceitful	**0.701**	0.022	-0.036	**0.525**
Dominance Item 9 Manipulative	**0.761**	-0.064	-0.036	**0.489**
Dominance Item 23 Insincere	**0.693**	0.003	0.039	**0.528**
Dominance Item 30 Garrulous	**0.649**	**-0.392**	-0.014	0.171
Emotional Item 5 Lacks Anxiety	**0.707**	-0.102	-0.033	0.003
Emotional Item 33 Lacks Pleasure	**0.412**	**0.513**	0.087	-0.036
Emotional Item 4 Lacks Emotional Depth	**0.598**	-0.036	**0.465**	0.046
Emotional Item 31 Lacks Emotional Stability	**0.718**	-0.112	-0.112	-0.170
Emotional Item 16 Lacks Remorse	**0.761**	0.035	0.035	0.213
Self Item 20 Self-centred	**0.812**	-0.043	-0.043	0.080
Self Item 14 Self-aggrandising	**0.816**	-0.298	-0.298	-0.006
Self Item 1 Sense of Uniqueness	**0.789**	**-0.425**	**-0.425**	0.035
Self Item 13 Sense of Entitlement	**0.734**	-0.148	-0.148	0.121
Self Item 22 Sense of Invulnerability	**0.788**	-0.310	-0.310	-0.020
Self Item 2 Self-justifying	**0.671**	0.005	0.005	0.219
Self Item 21 Unstable Self-concept	**0.369**	-0.123	-0.123	-0.066

For each of the CAPP items, the general factor explained a considerable proportion of the variance. Specifically, CAPP item loadings ranged from 0.369 (*Unstable self concept*) to 0.842 (*Domineering*); or in other words, the latent psychopathy factor explained 44.71% to 68.22% of the variance in these items. The items in which the latent psychopathy factor predicted the most variance were from the attachment and dominance domains, while those that loaded the least meaningfully on this factor were those from the behavioural domain, with the exception of *Aggressive*. Examination of the factor loadings for the three bi-factors suggested that they mirror aspects of emotional detachment, disinhibition, and deceitfulness. This is a close parallel to what has been found in previous research [30, 35]. Convergence between Spanish expert prototypicality ratings of CAPP symptoms [28] and their relative rank-ordered general factor loadings was calculated using Pearson’s correlation. The correlation coefficient was 0.824, p < 0.001. The equivalent comparison was conducted with Sellbom´ s general factor loadings, and for this analysis the correlation coefficient was 0.531, p = 0.001.

Another CFA study was applied to the CAPP dimensions. First all items were used (Table 7).

**Table 7:** Confirmatory factor analysis of CAPP dimensions with all items.

			Maximum likelihood			Weighted Least Squares		
	N	Df	CFI	TLI	RMSEA	CFI	TLI	RMSEA
**Attachment**	204	2	0.969	0.907	0.186	0.936	0.807	0.166
**Behavioural**	204	9	0.837	0.729	0.272	0.808	0.681	0.166
**Cognitive**	204	5	0.605	0.210	0.397	0.738	0.477	0.249
**Dominance**	204	9	0.919	0.866	0.174	0.843	0.738	0.137
**Emotional**	204	5	0.843	0.686	0.202	0.755	0.511	0.206
**Self**	204	14	0.935	0.902	0.133	0.867	0.801	0.103

CAPP: Comprehensive Assessment of Psychopathic Personality; df: degrees of freedom; CFI: Comparative (Fit) Index; TLI: Tucker-Lewis Index; RMSEA: Root Mean Square Error of Approximation.

Remembering that RMSEA can be artificially high for models with low degrees of freedom, best performance was shown by the attachment domain, followed by self and dominance. Poor fit was found for emotional, behavioural, and cognitive. A CFA was performed again removing items from the domains in the search for fit improvement. Best fit was found (Table 8) for the following item removals: Attachment (no item removed), behavioural (item 32 aggressive removed; still a poor fit), cognitive (items 28 lacks concentration and 29 lacks planfulness removed), dominance (items 11 antagonistic and 12 domineering removed), emotional (items 31 lacks emotional stability and 33 lacks pleasure removed) and self (items 2 self-justifying and 21 unstable self-concept removed).

**Table 8:** Confirmatory factor analysis of CAPP dimensions deleting items for best fit.

				Maximum likelihood			Weighted Least Squares		
		n	df	CFI	TLI	RMSEA	CFI	TLI	RMSEA
**No changes**	Attachment	204	2	0.969	0.907	0.186	0.936	0.807	0.166
**Removed item 32**	Behavioural	204	5	0.842	0.684	0.341	0.873	0.746	0.169
**Removed items 28 & 29**	Cognitive	204	0	1	1	0	1	1	0
**Removed items 11 & 12**	Dominance	204	2	1	1.03	0	1	1.06	0
**Removed items 31 & 33**	Emotional	204	2	1	1	0	1	1	0
**Removed items 2 & 21**	Self	204	5	0.986	0.972	0.095	0.968	0.985	0.077

CAPP: Comprehensive Assessment of Psychopathic Personality; df: degrees of freedom; CFI: Comparative (Fit) Index; TLI: Tucker-Lewis Index; RMSEA: Root Mean Square Error of Approximation.

Considering all the items, the attachment, self, and dominance domains showed the best fit and the emotional, behavioural, and cognitive domains the worst. When symptoms are removed trying to improve the fit of the different CAPP dimensions, the robustness of the attachment dimension and its symptoms becomes clear. It also becomes clear that the behavioural dimension is the least robust of all. The CFA, PCA (Table 9), and bi-factor data (Table 6) also demonstrated that the aggressive item is less related to the rest of the behavioural items. The fit of all other dimensions clearly improved with the removal of some of their items. In the cognitive domain, the removal of low prototypical symptoms such as lacks concentration and lacks planfulness suggest that these symptoms are of low interest for this dimension. The same seems to happen for unstable self concept, which is also less related to component 2 compared with the rest of the self items in the PCA analysis (Table 9), and it also loaded very low in the general factor from the bi-factor analysis. The fit increase from removing the self-justifying item, a highly prototypical item, suggests that all other items are more related to the grandiose and arrogant interpersonal style showing more homogeneous scores. In the emotional domain, the removal of the low prototypical lacks pleasure items [23, 24, 28] is clear. Lacks emotional stability, another emotional item, in the PCA analysis (Table 9) is less related to component 4 than the other emotional items but more related to component 1, as is unstable self concept. These data suggest that this item is not related to the callous and unemotional (CU) trait as the other emotional items are. Like unstable self concept, it seems to be more related to the behavioural symptoms and the pervasive impulsive conduct trait that, in the CAPP model, seems to be the least robust and prototypical [23, 24, 28]. Finally, regarding the dominance domain, the clear fit improvement found with removal of the highly prototypical [23, 24, 28] antagonist and domineering items that seem to be central for this dimension is controversial. In the PCA, these two items are less related than the others to component 2. Thus, we can suggest that all other items are more related to the grandiose and arrogant interpersonal style once again showing more homogeneous scores compared with these other two. This point of view is strengthened by the bi-factor analysis, that the deceitfulness bi-factor being constituted by these other three items (deceitful, insincere, and manipulative).

**Table 9:** CAPP rotated components matrix.

CAPP Items	Components					
	1	2	3	4	5	6
Attachment Item 18 Detached	-.028	.132	.211	**.847**	.184	.042
Attachment Item 8 Uncommitted	.391	.345	.243	**.557**	.270	-.076
Attachment Item 25 Unempathic	.105	.309	.510	**.580**	.171	-.199
Attachment Item 24 Uncaring	.409	.351	.484	**.468**	.101	-.178
Behavioural Item 3 Lacks Perseverance	**.834**	.234	.159	.122	-.124	-.144
Behavioural Item 26 Unreliable	**.774**	.409	.200	.090	-.055	-.167
Behavioural Item 15 Reckless	**.780**	.104	.048	.007	.354	.013
Behavioural Item 6 Restless	**.685**	.008	.126	-.023	.519	.163
Behavioural Item 17 Disruptive	**.763**	.144	.146	.159	.384	-.054
Behavioural Item 32 Aggressive	**.310**	.075	.566	.323	.428	-.144
Cognitive Item 19 Suspicious	-.041	.334	.132	.689	.179	.193
Cognitive Item 28 Lacks concentration	.836	.061	.076	-.003	-.020	.053
Cognitive Item 7 Intolerant	.135	.303	.790	.246	.091	.069
Cognitive Item 27 Inflexible	.197	.203	.722	.303	.107	.163
Cognitive Item 29 Lacks planfulness	.872	.144	.052	.164	-.007	.034
Dominance Item 11 Antagonistic	.268	.247	.732	.251	.119	-.051
Dominance Item 12 Domineering	-.029	**.320**	.645	.317	.430	-.036
Dominance Item 10 Deceitful	.186	**.784**	.149	.278	.051	-.135
Dominance Item 9 Manipulative	.214	**.780**	.201	.226	.161	-.108
Dominance Item 23 Insincere	.283	**.762**	.017	.325	.095	-.075
Dominance Item 30 Garrulous	.171	**.649**	.316	-.201	.364	-.041
Emotional Item 5 Lacks Anxiety	.202	.358	.178	**.324**	.551	-.186
Emotional Item 33 Lacks Pleasure	.183	.064	.393	**.558**	-.329	.256
Emotional Item 4 Lacks Emotional Depth	.120	.156	.254	**.731**	.004	-.157
Emotional Item 31 Lacks Emotional Stability	.607	.216	.411	.145	.280	.291
Emotional Item 16 Lacks Remorse	.185	.595	.227	**.384**	.197	.166
Self Item 20 Self-centred	.203	**.503**	.223	.409	.437	.127
Self Item 14 Self-aggrandising	-.042	**.538**	.468	.108	.481	.095
Self Item 1 Sense of Uniqueness	.035	**.578**	.293	.080	.573	.034
Self Item 13 Sense of Entitlement	.117	**.646**	.457	.045	.148	.226
Self Item 22 Sense of Invulnerability	.221	**.422**	.218	.212	.657	.073
Self Item 2 Self-justifying	.159	**.622**	.225	.287	.056	.248
Self Item 21 Unstable Self-concept	.669	.081	.096	-.007	-.014	.500

CAPP: Comprehensive Assessment of Psychopathic Personality.

A principal components analysis (PCA) was run on the CAPP items (Table 9). The suitability of PCA was assessed prior to analysis. Inspection of the correlation matrix showed that all variables had at least one correlation coefficient greater than 0.4. The overall Kaiser-Meyer-Olkin (KMO) measure was 0.937 with individual KMO measures all greater than 0.9. According to Kaiser´s categories, they can be classified as marvellous, indicating that there is adequacy of sampling. The results of the Bartlett's test of sphericity showed that it was statistically significant (p < 0.001), indicating that the data were likely factorable.

PCA revealed five components that had eigenvalues greater than one and that explained 44.71%, 12.10%, 6.70%, 4.71%, and 3.76% of the total variance, respectively. Visual inspection of the scree plot indicated that six components should be retained. In addition, and as items are classified in six dimensions, a six-component solution was considered to meet the interpretability criterion, and therefore six components were retained.

The six-component solution explained 74.57% of the total variance. A Varimax orthogonal rotation was employed to aid interpretability (Table 9). The rotated solution exhibited 'simple structure. The interpretation of the data was consistent with previous studies that showed a clear relationship between the attachment and emotional domains and also between dominance and self [30]. It should be remembered that, although we can establish that the behavioural component loads mainly on component number 1, it is not clear enough on which component the largest part of the items of the cognitive dimension load.

The PCA analysis shows a strong association in component 4 (Table 9) between the attachment items and all emotional ones, with the exception of lacks emotional stability. The suspicious cognitive item is also associated with this component and is also part of the detachment bi-factor.

Another strong association was found in component 2 (Table 9), between all dominance items, with the exception of the antagonistic item and all self items, with the exception of the unstable self-concept item.

All behavioural items, two cognitive ones (lacks concentration and lacks planfulness), and lacks emotional stability and unstable self-concept are strongly associated in component 1.

In component 3, the antagonistic and domineering dominance items are associated with the unempathic and uncaring attachment items, with the aggressive behavioural item and with the intolerant and inflexible cognitive items. This component seems to be more related to aggression.

## Discussion

The purpose of this study was to analyse CAPP scores in a large set of participants from a non-convenience sample following standard assessment. To our knowledge, this is the first time a non-convenience sample from a non-English- speaking country in Southern Europe has been assessed with the CAPP.

Internal consistency assessed with Cronbach´ s α and with other reliability indexes from Classical Test Theory for the CAPP total score, domains, and individual items was excellent (Tables 2 and 3). Likewise, consistency correlation between domains was also high, not only with the total score as expected, but also with one another (Table 4). As in prototypicality studies [23, 24, 28], the behavioural dimension and its items seemed to be less connected to the other domains and their items, with the exception of the cognitive domain (0.75 correlation was found), which was also found to be less prototypical in aforesaid studies.

In keeping with previous prototypicality research [23, 24, 28] CFA analysis of the CAPP dimensions and PCA analysis reinforce the correlation data and show how CAPP items are organized around three main traits: (1) detached–unemotional, (2) interpersonal: dominant, narcissistic, and aggressive, and (3) behaviourally disinhibited. This reflects the pattern found in the Psychopathy Checklist -revised [4].

As in previous research [30, 35] bi-factor analysis indicates that these main traits are arranged around a robust higher order factor that mirrors general psychopathy, maintaining three sub-factors: detachment, disinhibition, and deception. Supporting the correlation and the CAPP dimensions CFA analyses, a rank order of the latent factor loadings observed for the general psychopathy factor shows the highest loadings for dominance, attachment, and self items, and the lowest for behavioural ones, in keeping with previous prototypicality research [23, 24, 28].

Putting all the analyses together, we arrive at a broad general construct of psychopathy with specific subfactors constituted by particular subsets of items. This general psychopathy factor and its residual factors are configured by a constellation of three distinctive traits: detachment, interpersonal dominance, and disinhibition. This three-factor model fits nicely with the three traits that experts consider essential for the diagnosis of psychopathy: callous and unemotional (CU) affects reflecting a deficient affective experience (components 4 and 3, and one bi-factor), a grandiose and arrogant interpersonal style (component 2, and one bi-factors), and a pervasive impulsive conduct (component 1 and one bi-factor) [1–4]. This three- factor model of psychopathy has also been found in previous CAPP research conducted in a large international sample of community-dwelling participants who performed CAPP self-ratings [30] This robust relationship between self-rating and clinician-rating scores in different samples shows the robustness of the CAPP for detecting the core traits of psychopathy without assessing criminality [25, 36] PCA analysis and bi-factor analysis also point to why no good fit was found in the CFA analysis for CAPP dimensions. Many of the CAPP items do not fit in just one component, and the finding of a general factor in the bi-factor analysis reinforces this point of view. Combining the PCA, bi-factor, and CAPP dimensions CFA analyses we found a clear relation with the results from prototypicality studies. This data convergence indicates that the removal of less prototypical items improves the fit of many of the dimensions, and that the less prototypical and robust dimension and items are related to the pervasive impulsive conduct trait. All this would suggest that the other two traits, callous and unemotional (CU) affects, reflecting a deficient affective experience and a grandiose and arrogant interpersonal style are more central for the CAPP model, and probably also for the concept of PPD. Without their less prototypical items more related to the behavioural dimension, the rest of the dimensions including the aggressive item are organized around the CU and the grandiose and arrogant interpersonal style traits, with some inmates being more into aggression and others into deception. Once again, but this in the current research using clinician-ratings, substantial convergence of relative importance of CAPP items and dimensions with the construct of psychopathy using several highly divergent methodologies has been proven [30].

Univariate and multivariate analyses indicated that CAPP total score was more relate to personality psychopathology assessed with the IPDE, especially with paranoid, narcissistic and antisocial personality disorders, than with criminal behaviour and drug use. IPDE borderline also showed a significant relation with CAPP total score in both types of analysis, but did not show a good correlation with the attachment and dominance domains, which are key domains for the psychopathy construct when assessed with the CAPP. Although less associated with CAPP total score, IPDE histrionic also showed significant correlations with all CAPP dimensions except with attachment, dominance and emotional. All these point to the fact that, in general, from a Diagnostic and Statistical Manual of Mental Disorders (DSM) point of view, CAPP dimensions do correlate with cluster B personality disorders and paranoid personality disorders [37]. Cluster C and Cluster A schizophrenia-related personality disorders do not show a strong significant association with CAPP dimensions and total score. But to be highly psychopathic from a CAPP perspective a Cluster B or paranoid diagnosis is not enough, inmates must also fit the three factor model and for doing so they need to score high on the dominance and attachment domains.

So, as intended by the authors who developed it, the CAPP is clearly associated with the psychopathology of personality, especially cluster B, without a strong association with criminal activity [25, 36] Not finding a significant relation between number of official charges and CAPP scores (total and domains) is evidence of this weak association. Furthermore, the CAPP does not seem to be heavily influenced by drug abuse, a clear risk factor for criminal activity [38]. It is noteworthy that disorderly conduct, driving while intoxicated, and major traffic violations, three of the four official charges that showed a significant association with CAPP total score only in the univariate analysis (Table 1) are more related to impulsivity than to instrumental criminal activity. However, the results of the current study cannot fully answer whether or not criminal behaviour is significantly associated or not with the CAPP, and further studies with other samples and other criminal variables are needed [39–43].

### Limitations

This study is not without limitations. Even though the researchers were scrupulous about blinding procedure, it could have been broken by the inmates during assessment. This is an inevitable limitation, as inmates have to be assessed in a semi-structured way that enables them to break the blind design. With only one researcher doing the CAPP and IPDE assessment and scoring, the study´ s internal validity can be enhanced but the external validity can be diminished, as that researcher may be biased by his own theoretical ideas. The IPDE personality disorders prevalence variance in the current study compared with previous research could be a sign of this bias [32] moreover, personality disorder prevalence rates vary widely among studies [32]and, in many of them, rates very close to the ones in our study were found [44]. This was especially relevant for antisocial personality disorder, for which we applied DSM-IV Criterion C (*there is evidence of conduct disorder with onset before age 15*) very strictly. With this strategy we reduce the risk of false positives related to drug abuse but false negatives may have been included.

Further research is needed to replicate these findings and to study the association between CAPP scores and the risk of criminal and violent recidivism associated with PPD.

## Conclusions

The current study sought to evaluate psychometric properties and construct validity of the CAPP in a non-convenience sample of participants. In this sample, as in previous prototypicality research, the CAPP showed robust psychometric properties, and its construct validity proved to be good, as it was capable of discriminating among the three psychopathic traits without relying on the assessment of criminal behaviour. Callous and unemotional (CU) affects reflecting deficient affective experience and grandiose and arrogant interpersonal style-related items and dimensions seemed to be more central for the CAPP model. CAPP assessment of psychopathy through clinician-ratings in a correctional setting maintains the validity found in previous non-clinical research; therefore it is a valid and promising tool for routine evaluation of psychopathic traits in forensic, clinical, and correctional settings.
